# Beyond the tear: the enduring role of aortic pathology in the era of genomic medicine

**DOI:** 10.1136/openhrt-2026-004003

**Published:** 2026-03-19

**Authors:** Nimrat Grewal, Hans W M Niessen

**Affiliations:** 1 Department of Cardiothoracic Surgery, Amsterdam University Medical Centres, Amsterdam, The Netherlands; 2 Department of Cardiothoracic Surgery, Yale School of Medicine, Yale, New Haven, USA; 3 Department of Pathology, Amsterdam University Medical Centres, Amsterdam, The Netherlands

**Keywords:** Aneurysm, Dissecting, Aortic Aneurysm, Marfan Syndrome, Genetics

## Abstract

Thoracic aortic dissection is often approached as an acute and localised event, and pathological examination has traditionally focused on the dissected segment. In daily practice, however, most clinicians recognise that the dissection itself is rarely the starting point of disease. Structural abnormalities of the aortic wall are frequently present long before rupture occurs and may extend well beyond the site of failure.

At the same time, the diagnostic landscape of thoracic aortic disease is changing rapidly. Advances in genetic and molecular techniques have increased the detection of potentially disease-causing variants, but their clinical interpretation remains challenging. In this setting, histopathological examination of the aortic wall provides essential phenotypic context and continues to play a key role in recognising and interpreting genetic disease. The value of pathology, however, depends strongly on the representativeness of the sampled tissue.

In this Brief Communication, we discuss why routine reliance on dissected aortic tissue may be insufficient to characterise the underlying disease process. We argue for a more deliberate approach to tissue selection, with attention to macroscopically intact aortic segments, and highlight the importance of standardised reporting and appropriate biobanking infrastructure. Despite ongoing advances in genomic medicine, careful pathological examination of the aorta remains a cornerstone in understanding thoracic aortopathy.

## Introduction

 Thoracic aortic dissection, defined as a tear in the largest vessel, is usually encountered as an acute and clearly localised event. From a surgical point of view, the problem appears well defined: a segment of aorta has failed and requires replacement.^1^ Pathological examination has traditionally followed this logic. The dissected segment, readily available and clinically central, is most often submitted for histological assessment.^2^

At the same time, thoracic aortic dissection is generally considered a clinical presentation of pre-existing aortic wall pathology.^3^ In many patients, whether they ultimately prove to have a heritable disorder or not, structural abnormalities of the aortic wall are present long before the acute event.^4^,^6^ These abnormalities are typically silent and diffuse, only becoming clinically apparent once mechanical failure occurs.^7^

This discrepancy between how thoracic aortic disease develops and how tissue is routinely sampled has received surprisingly little attention. While surgery and pathology are understandably focused on the point of failure, our biological understanding of thoracic aortopathy increasingly emphasises chronic wall disease rather than focal catastrophe.^8^ The question of whether the tissue we routinely examine is actually suited to represent that disease is often left unaddressed.

In recent years, this issue has gained additional relevance. Pathological findings are no longer used solely for descriptive diagnosis but increasingly feed into genetic evaluation, risk stratification and research infrastructures. In that context, the representativeness of the sampled tissue matters. This paper revisits the role of pathological examination of the thoracic aorta in the current era and argues that, despite rapid advances in genomic technologies, pathology remains a cornerstone in recognising and interpreting genetic disease when tissue sampling and infrastructure are appropriately aligned.

### The dissected segment and its limitations

**Figure 1:**
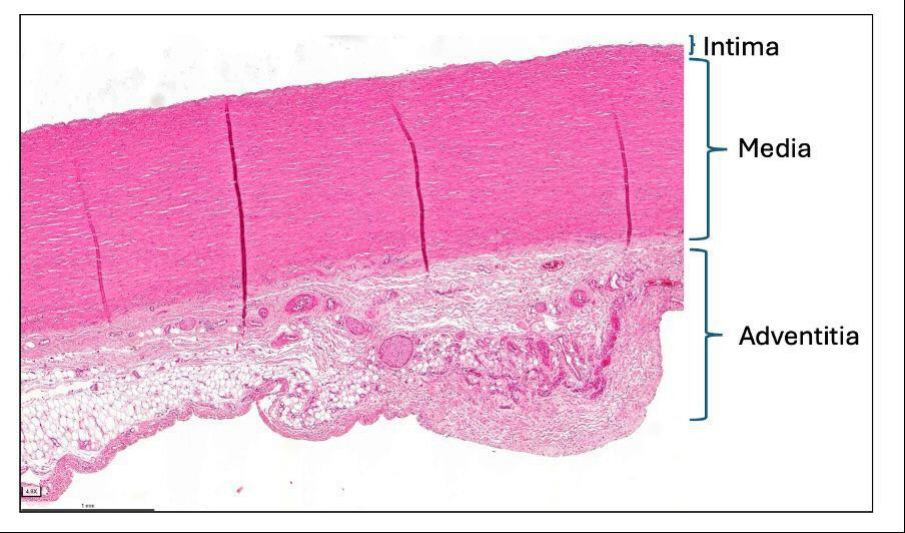
Macroscopically normal thoracic aorta. Transverse 5 µm section stained with H&E. The wall shows the expected layered organisation with a thin intima, a media composed of regularly oriented lamellar units and an adventitia containing connective tissue and vasa vasorum. Overall architecture is preserved without disruption of lamellar alignment.

Macroscopically intact thoracic aortic tissue shows preserved wall layering with a thin intima, a media composed of regularly arranged elastic lamellae with interspersed smooth muscle cells, and an adventitia containing connective tissue and vasa vasorum. In this setting, lamellar alignment and cellular distribution can be appreciated without distortion (figure 1).

**Figure 2:**
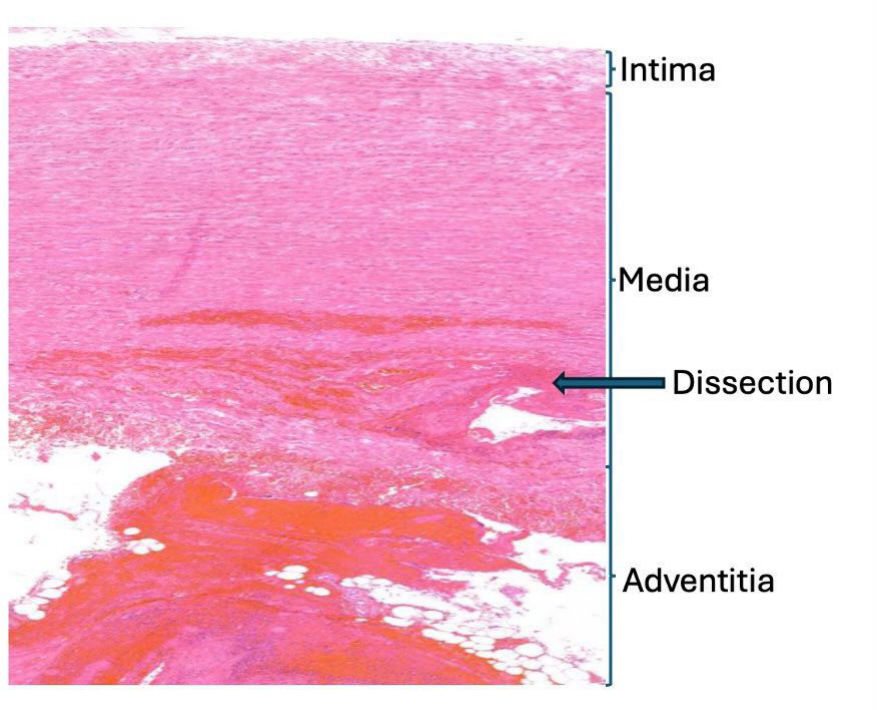
Representative histological section of the dissected thoracic aorta. Five-micrometre transverse histologic section stained with H&E. The section demonstrates the typical features of acute aortic dissection, including disruption of the medial lamellar architecture, intramural haemorrhage, oedema and inflammatory cell infiltration. The dissection plane traverses the aortic wall, resulting in marked structural distortion and loss of normal tissue orientation. As a consequence, assessment of pre-existing medial abnormalities—such as elastin fragmentation, extracellular matrix organisation and vascular smooth muscle cell morphology—is inherently challenging in this setting. This illustrates how acute dissection-related changes may dominate the histological picture and complicate interpretation of the underlying chronic disease process.

From a pathological perspective, the dissected segment is a difficult substrate. By definition, it is a tissue that has undergone acute mechanical failure. Intramural haemorrhage, lamellar disruption, oedema and inflammatory changes are almost invariably present.^9^ These features are clinically relevant, but they also dominate the histological picture (figure 2).

**Figure 3:**
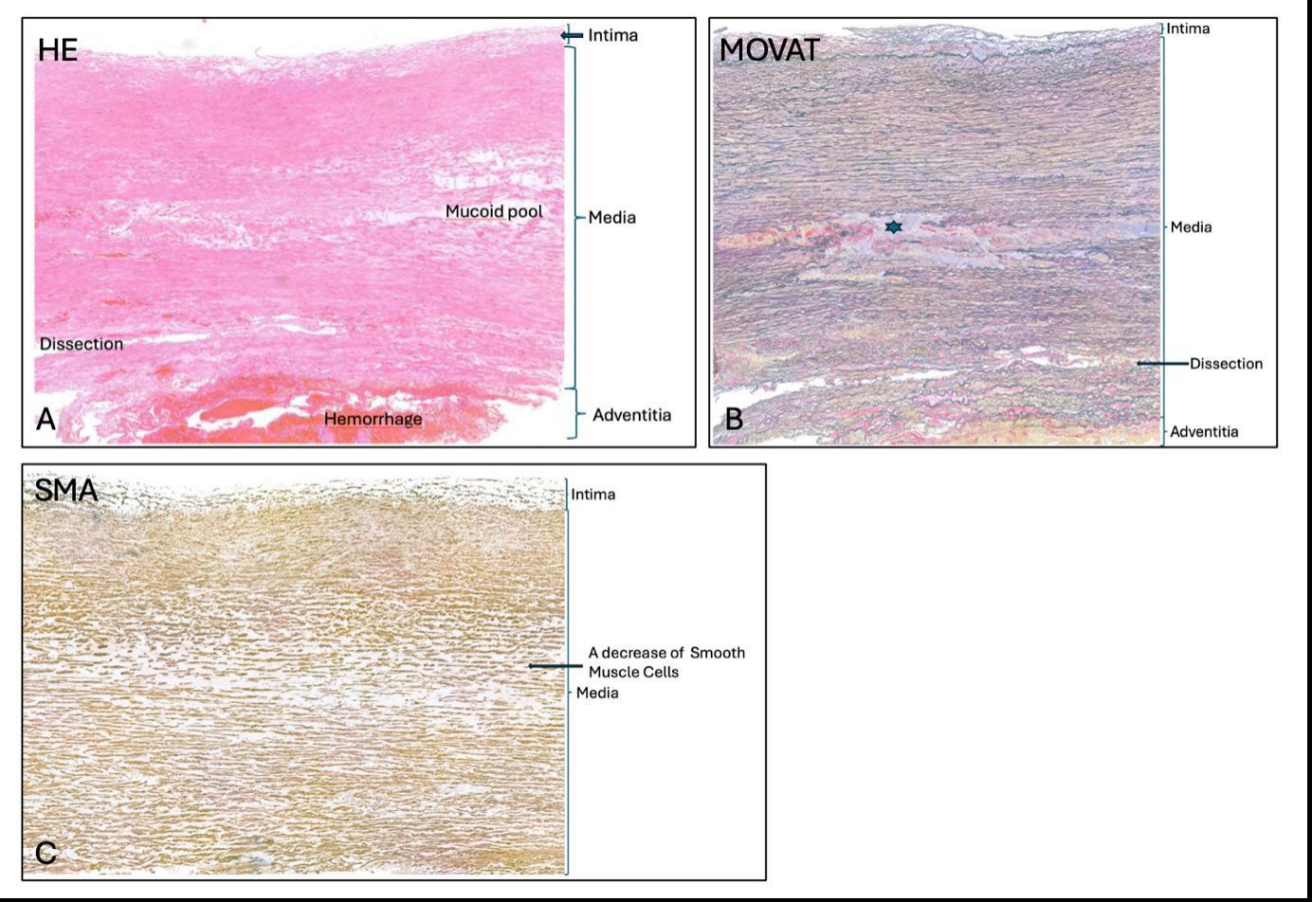
Representative histological sections of a macroscopically intact thoracic aortic segment adjacent to the dissection plane. All panels show five-micrometre transverse histologic sections obtained adjacent to, but not through, the dissection plane. (**A**) H&E staining demonstrating preserved overall wall architecture with recognisable intima, media and adventitia. Within the media, areas of mucoid extracellular matrix accumulation are present, while acute dissection-related haemorrhage is absent, allowing clearer assessment of chronic medial changes. (**B**) Movat pentachrome staining of the same region highlights medial structural organisation and extracellular matrix composition. Disruption and focal loss of elastic fibres are visible (asterisk), while the dissection plane remains spatially separated from the area of interest, preserving tissue orientation. (**C**) α-smooth muscle actin (α-SMA) immunohistochemistry demonstrating reduced smooth muscle cell content within the media, consistent with chronic medial degeneration rather than acute injury. Together, these sections illustrate how macroscopically intact aortic tissue adjacent to the dissection plane allows more reliable evaluation of elastin architecture, smooth muscle cell integrity and matrix alterations than tissue dominated by acute dissection-related changes.

In this setting, it becomes challenging to assess more subtle, pre-existing abnormalities of the aortic wall. Elastin fragmentation, alterations in collagen organisation, changes in proteoglycan content or variations in vascular smooth muscle cell density may all be present (figure 3), but it is often unclear whether these reflect chronic disease or are secondary to the dissection itself. Surgical manipulation and ischaemic changes further complicate interpretation.

In daily practice, this ambiguity is usually accepted. The dissected tissue is what is available, and the pathology report reflects that reality. Problems arise, however, when histological findings are later revisited in a broader diagnostic or research context. When pathology is used to support or question a suspected heritable aortopathy, or to help interpret uncertain genetic findings, the limitations of dissected tissue become more apparent. In such cases, the pathology does not necessarily answer the question being asked, not because of lack of expertise, but because of the nature of the tissue itself.

### Thoracic aortopathy beyond the site of rupture

There is increasing recognition that thoracic aortic disease is neither focal nor uniform. Histopathological abnormalities have been described throughout the thoracic aorta, including in segments that appear macroscopically normal at the time of surgery.^10^ Disease severity may vary considerably within the same patient, both along the length of the aorta and around its circumference. More importantly, no difference in pathology has been encountered between the convex and concave side of the aortic wall, with the convex (outer) wall representing the typical site of jet impingement.^11 12^

Importantly, macroscopically intact tissue is not synonymous with normal tissue. On the contrary, non-dissected segments often provide clearer insight into the underlying disease process. Elastin architecture is better preserved, allowing fragmentation patterns to be appreciated. Changes in smooth muscle cell organisation or loss are easier to distinguish from acute injury. Matrix alterations that would be difficult to recognise in disrupted tissue may become evident (figure 3).

These observations have practical implications. If thoracic aortopathy is a diffuse and heterogeneous process, then sampling strategies focused exclusively on the site of dissection inevitably provide an incomplete picture. They preferentially capture the endpoint of disease rather than its substrate.

### Pathology in the era of genetic and advanced molecular techniques

The expanding availability of whole-exome and whole-genome sequencing has transformed the diagnostic work-up of thoracic aortic disease. In this setting, it may be tempting to regard histopathology as secondary to molecular analysis. In practice, the opposite is often true.

Pathological assessment frequently shapes the clinical suspicion that prompts genetic testing in the first place.^9^ Patterns of medial degeneration, the presence or absence of inflammation and the overall organisation of the aortic wall all influence whether a heritable disorder is considered likely.^13^ Even after genetic testing has been performed, pathology remains central, particularly when variants of uncertain significance are identified. In such cases, histological findings may support a causal role for a variant or, conversely, argue against it. Blood-based analyses and genetic testing provide complementary information in thoracic aortic disease, but circulating markers remain exploratory and do not reflect local wall structure. In practice, histopathological assessment of resected tissue may represent the first detailed phenotypic evaluation and can lead to referral for genetic consultation in selected patients.

These interactions underscore that pathology is not rendered obsolete by genomic technologies. Rather, its role evolves. Pathology provides the phenotypic context in which genetic findings are interpreted. Without that context, molecular data risk becoming detached from biological reality.

However, this role can only be fulfilled if the examined tissue reflects the chronic disease process rather than acute failure. The value of pathology in recognising genetic disease is therefore directly linked to how and where tissue is sampled.

### Rethinking what we send to pathology

Against this background, it may be time to reconsider routine tissue submission practices in thoracic aortic surgery. This is not a call for rigid protocols, but for greater awareness of the consequences of sampling choices.

Whenever surgically feasible, submission of a macroscopically intact thoracic aortic segment allows for more reliable assessment of medial architecture and chronic pathological changes. Tissue obtained contralateral or adjacent to the dissection plane is often less affected by acute disruption and therefore more informative with respect to the underlying disease. Dissection-adjacent tissue may still be valuable, particularly for studying mechanisms of wall failure, but it should not be the only material available for evaluation.

Equally important is clarity in reporting. Consistent attention to key features, such as elastin organisation, smooth muscle cell characteristics and matrix composition, facilitates comparison across patients and supports integration with genetic data.

### Beyond sampling: the need for infrastructure

The value of pathological examination does not depend on sampling alone. It also depends on how tissue, data and expertise are organised. As interest in biobanking and translational research grows, the aorta is increasingly sampled not only for diagnostic purposes but also for future analyses.

This requires infrastructure. Clear pathways for tissue handling, storage, annotation and linkage to clinical and genetic data are essential. Without such structures, valuable biological material risks being underutilised or lost. Conversely, well-organised biobanks enable pathology to remain a living component of patient care and research rather than a static report in the medical record.

In this context, surgeons, pathologists and geneticists are jointly responsible. Decisions made in the operating room influence what can later be studied, interpreted and translated. Recognising this interdependence is a necessary step towards more integrated care and research in thoracic aortic disease.

## Conclusion

Thoracic aortic dissection is a dramatic clinical event, but it represents only one moment in the course of a chronic and often diffuse disease. In an era of rapidly advancing genetic and molecular techniques, pathological examination of the aorta remains a cornerstone in recognising and interpreting underlying disease mechanisms, including genetic abnormalities. Its continued relevance, however, depends on deliberate tissue selection and appropriate infrastructure.

By paying closer attention to which tissue is examined and how pathological data are embedded within broader diagnostic and research frameworks, clinicians can ensure that pathology continues to inform, rather than merely document, thoracic aortic disease.
